# Prenatal diagnosis of trisomy 8 mosaicism, initially identified by cffDNA screening

**DOI:** 10.1186/s13039-022-00616-y

**Published:** 2022-09-01

**Authors:** Junjie Hu, Kai Yan, Pengzhen Jin, Yanmei Yang, Yixi Sun, Minyue Dong

**Affiliations:** 1 Women’s Hospital, Zhejiang University School of Medicine, 1 Xueshi Road, Hangzhou, 310006 Zhejiang Province China; 2 The Key Laboratory of Reproductive and Genetics, Ministry of Education, Hangzhou, China

**Keywords:** Trisomy 8 mosaicism, Cell-free fetal DNA (cffDNA), Noninvasive prenatal testing (NIPT), Rare autosomal trisomies, Prenatal diagnosis

## Abstract

**Background:**

So called cell-free fetal DNA (cffDNA) in the maternal plasma, which is derived from placenta, is widely used to screen fetal aneuploidies, including trisomy 21, 18, 13 and sex chromosomes. Here we reported a case of trisomy 8 mosaicism (T8M), which was initially identified via cffDNA screening in noninvasive prenatal testing (NIPT).

**Methods:**

A 35-year-old woman received cffDNA screening at 17th week of gestation. Amniocentesis was performed subsequently, and karyotyping, single-nucleotide polymorphism array (SNP-array) and BACs-on-Beads™ (BoBs™) were used to determine fetal chromosome content. Interphase fluorescence in situ hybridization (FISH) was applied to determine the copy number of chromosome 8.

**Results:**

An enhanced risk for fetal trisomy 8 was identified by cffDNA screening in the studied pregnant woman. After amniocentesis trisomy 8 was found in 1 of 73 metaphases. SNP-array on DNA derived from cultured amniocytes and neonatal cord blood cells suggested the presence of T8M. Interphase FISH on native neonatal cord blood cells confirmed T8M with a percentage of 10%. The Bobs™ fluorescence data also suggested that 8q23-8q24 was amplified.

**Conclusions:**

The current study shows that NIPT is suited to provide hints on rare autosomal trisomies, which have to be further validated and confirmed by other approaches.

## Introduction

Cell-free fetal DNA (cffDNA) in maternal blood plasma was first discovered in 1997 [1]. These are DNA fragments circulating in the maternal plasma mostly derived from the placenta [2]. cffDNA screening, also known as noninvasive prenatal testing (NIPT), is widely applied for detecting fetal chromosome abnormalities, especially trisomy 21, 13, 18 and sex chromosomes [3, 4], which are under the recommendation of the American College of Medical Genetics and Genomics (ACMG) and the American Congress of Obstetricians and Gynecologists (ACOG) [5].

Rare autosomal trisomies (RATs) refer to trisomies other than trisomy 21, 18 or 13 [6]. RATs are excluded from routine NIPT due to a lack of large-scale population data and low positive predictive values (PPTs). Trisomy 8 mosaicism (T8M), also known as Warkany syndrome, is a rare chromosomal disorder, usually caused by a post zygotic non-disjunction. With an estimated prevalence ranging from 1:25,000 to 1:50,000, it affects men more than women [7].

Here we reported a case of T8M initially identified by cffDNA screening. T8M was confirmed by single-nucleotide polymorphism array (SNP-array), BACs-on-Beads™ (BoBs™) and interphase fluorescence in situ hybridization (FISH) on the native amniocytes as well as neonatal cord blood, with a percentage of 10%.

## Materials and methods

### Case report

**Fig. 1:**
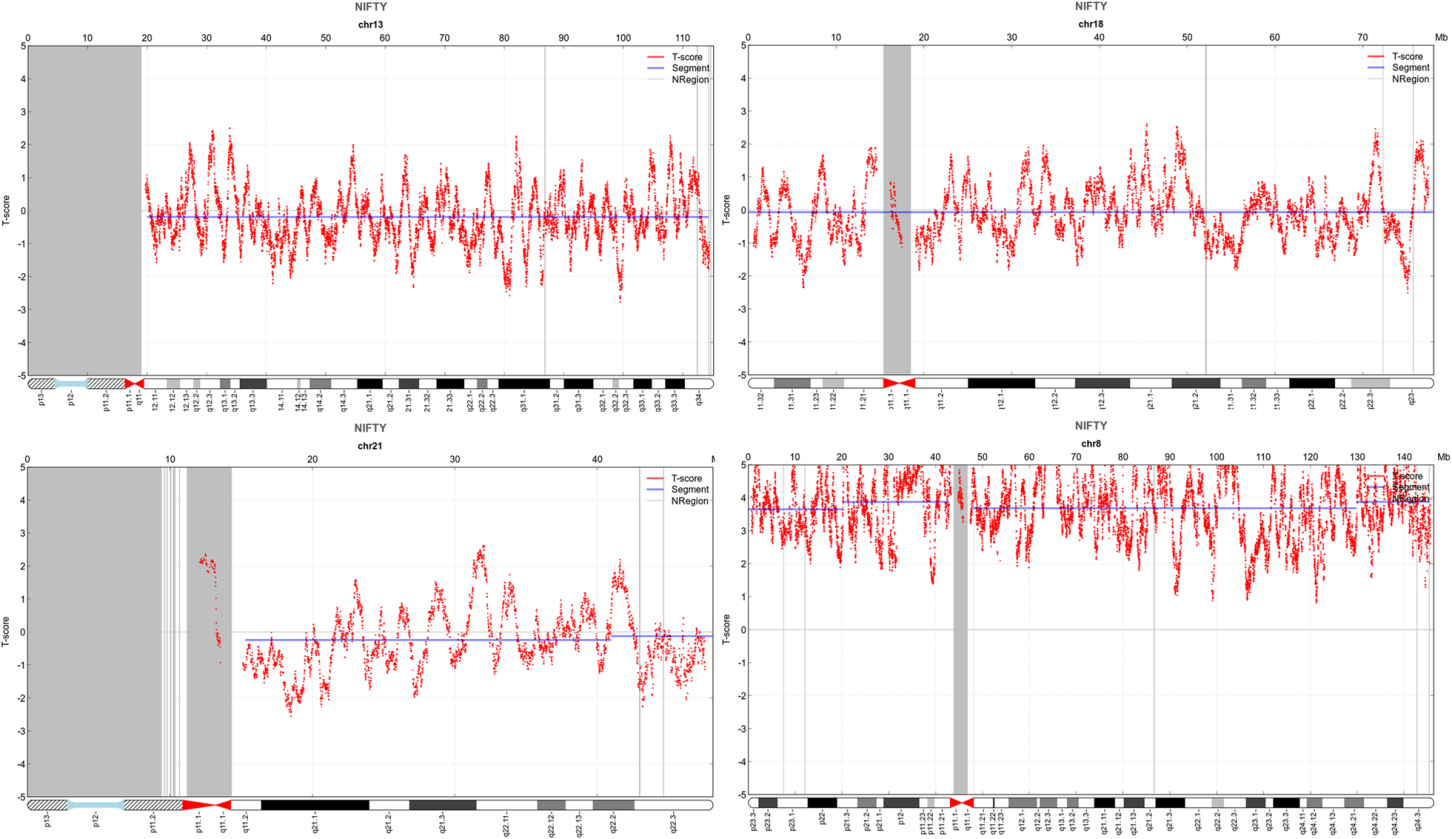
NIPT results of fetal chromosomes 13, 18, 21 and 8. The horizontal axis represents genomic location (Mb) and the vertical axis represents t-score. NIPT revealed the normal chromosomes (**A–C**) and an extra copy of chromosomes 8 (**D**)

**Fig. 2:**
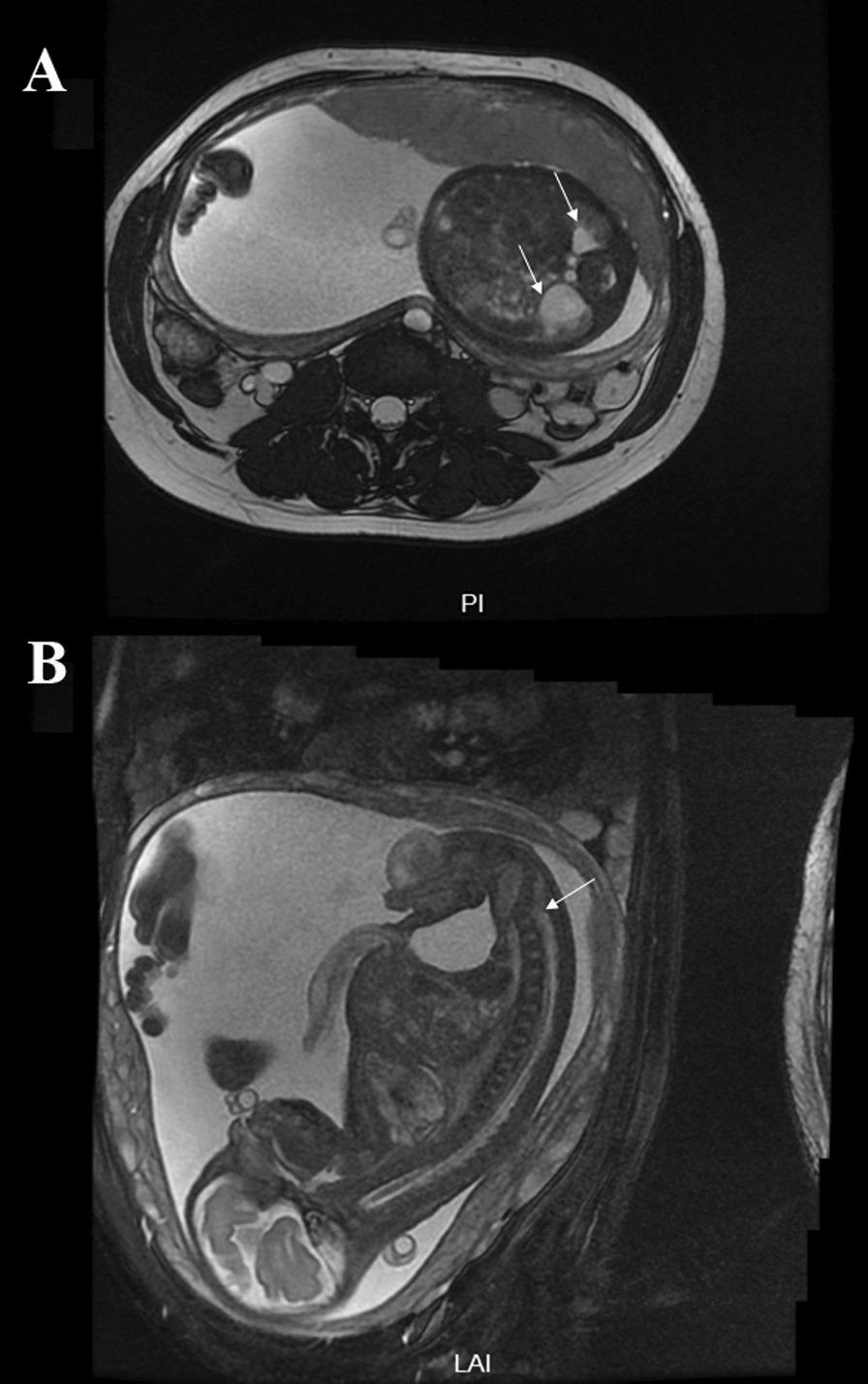
Magnetic resonance imaging. The fetus showed **A** hydronephrosis, **B** irregular spine in sagittal section

A 35-year-old healthy woman who had a singleton pregnancy referred to the Department of Reproductive Genetics, Women’s Hospital, School of Medicine, Zhejiang University. Peripheral blood sample was subjected to NIPT at 17th weeks of gestation. High risk of trisomy 8 was identified (Fig. 1). To confirm the results, amniocentesis was conducted at 23rd weeks of gestation.The fetal sample was further analyzed by karyotyping, SNP-array, FISH and Bobs™. In addition, hydronephrosis and irregular spine were observed in the fetal sonography and magnetic resonance imaging (MRI) at the 22th and 32th week of gestation, respectively (Fig. 2).

The Ethics Committee of Women’s Hospital, School of Medicine, Zhejiang University approved this study (IRB-20210170-R). All participants provided written informed consent.

### cffDNA screening

Maternal blood was collected in an EDTA-K_2_ containing tube. Plasma was separated via centrifugation at 1,600 g for 10 min at 4 °C. Then, the supernatant was re-centrifuged at 14,000 g for 10 min at 4 °C to remove cell debris. The subsequent procedures, including the isolation of cell-free DNA, library construction and sequencing, were performed according to the provider’s instructions (BGI, Shenzhen, China).

### Amniocentesis and fetal karyotyping

Amniocentesis was performed at the 23rd week of gestation under real-time ultrasound guidance. A total of 30 ml amniotic fluid was collected and the initial 5 ml was discarded. Amniocytes were cultured with BIOAMF-2 Complete Medium (Biological Industries, Cromwell, CT) in a 5% CO_2_ incubator at 37 °C. G-band analysis at 320–400 band resolution was performed on the cultured cells, according to the standard procedure.

### Single-nucleotide polymorphism array (SNP-array)

The amniocytes were cultivated for 7 days to exclude the visible maternal blood contamination. Fetal genomic DNA was extracted from the cultured amniocytes and neonatal cord blood cells using the Gentra Puregene Kit (Qiagen, Hilden, Germany). CytoScan™ HD array (Affymetrix, SantaClara, CA) was used to analyze the copy number, following the manufacturer’s instructions. The Chromosome Analysis Suite (ChAS) soft ware (Affymetrix, Santa Clara, CA) was used to analyze the raw data and visualize the results based on the GRCh37/hg19 assembly.

### Interphase FISH analysis

Neonatal cord blood cells were analyzed using trio-FISH with the TelVysion 8q Spectrum Red, Vysis CEP 4 Spectrum Auqa and TelVysion 2p Spectrum Green probes. Interphase spread hybridization and wash were performed following the manufacturer’s instructions (Vysis, Downers Grove, IL, USA). Interphase spreads were counterstained with4′, 6-diamidino-2-phenylindole (DAPI; Vysis, Downers Grove, IL, USA) and analyzed using a Zeiss Imager.A2 microscope (Zeiss, Marly-le-Roi, France). Subsequently, image acquisition was performed using a charge-coupled device camera with Isis (FISH Imaging System, MetaSystems, Altlussheim, Germany) [8].

### BACs-on-Beads™ (BoBs™) assay

DNA was extracted from the neonatal cord blood cells using the Gentra Puregene Kit (Qiagen, Hilden, Germany). BoBs™ assay was used to detect copy number changes of the 8q23-8q24 (Langer–Giedion syndrome) region. This assay was obtained from BoBs™ assay manufacturer (PerkinElmer, Wallac Oy, Finland), and the fluorescence data were analyzed with BoBsoft software (PerkinElmer, Wallac Oy, Finland).

## Results

### Identification of fetal trisomy 8 by cffDNA screening

cffDNA screening showed that low risk for trisomies 13, 18 and 21 (Fig. 1A–C). However, the t-score of chromosome 8 was as high as 4.5 (Fig. 1D), indicating that the fetus may carry an extra copy number of chromosome 8. Additionally, no sub-chromosomal deletion and duplication were found.

### Determination of trisomy 8 mosaicism

**Fig. 3:**
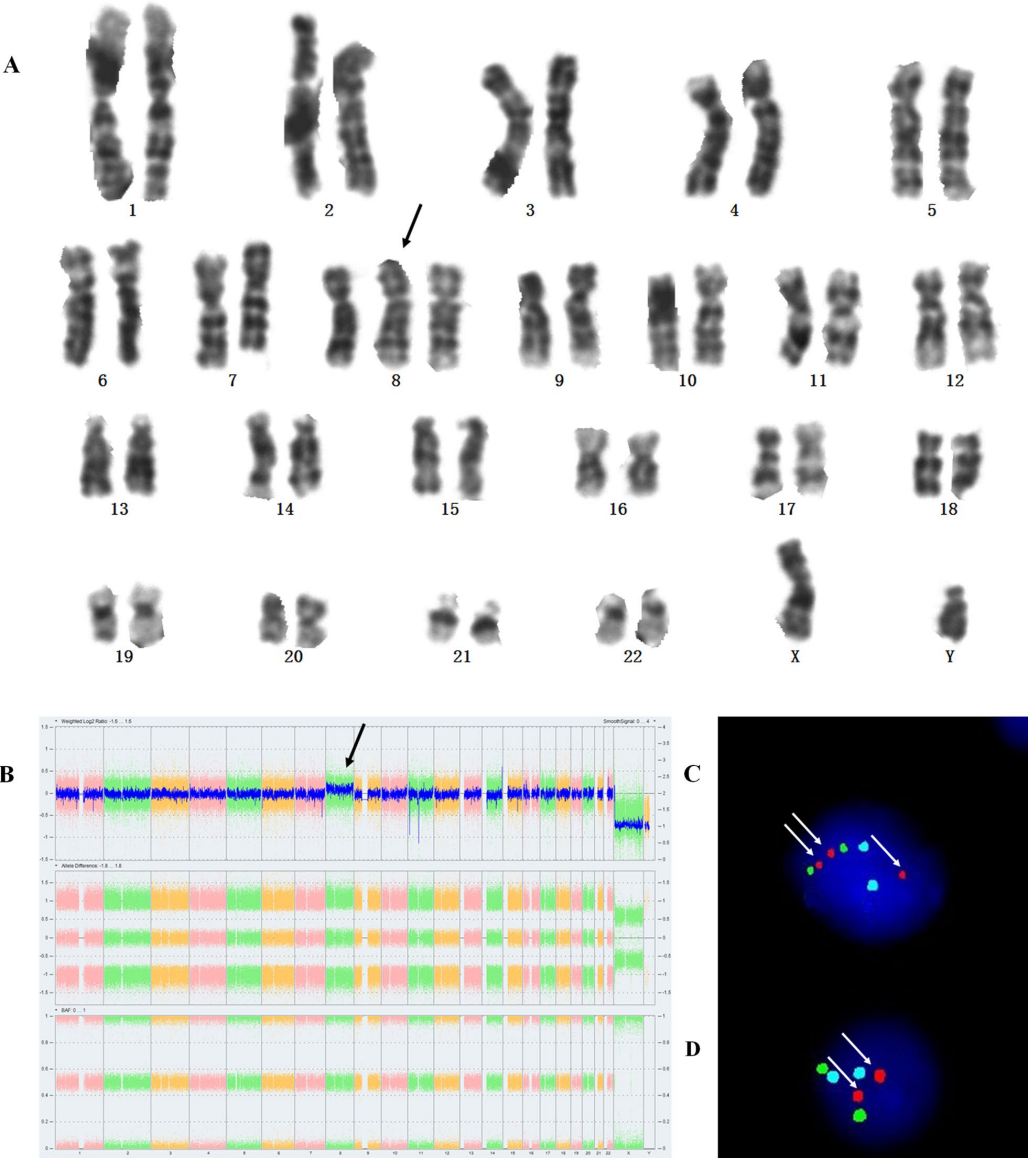
Karyotyping, SNP-array and FISH analysis of the fetus. **A** Karyotyping from cultured amniocytes. **B** SNP-array results from cultured amniocytes. **C**, **D** chromosome 8 (Spectrum Red), chromosome 4 (Spectrum Blue) and chromosome 2 (Spectrum Green) probes on the neonatal cord blood. There are three red signals in T8 cell (**C**) and two red signals in the normal cell (**D**)

**Fig. 4:**
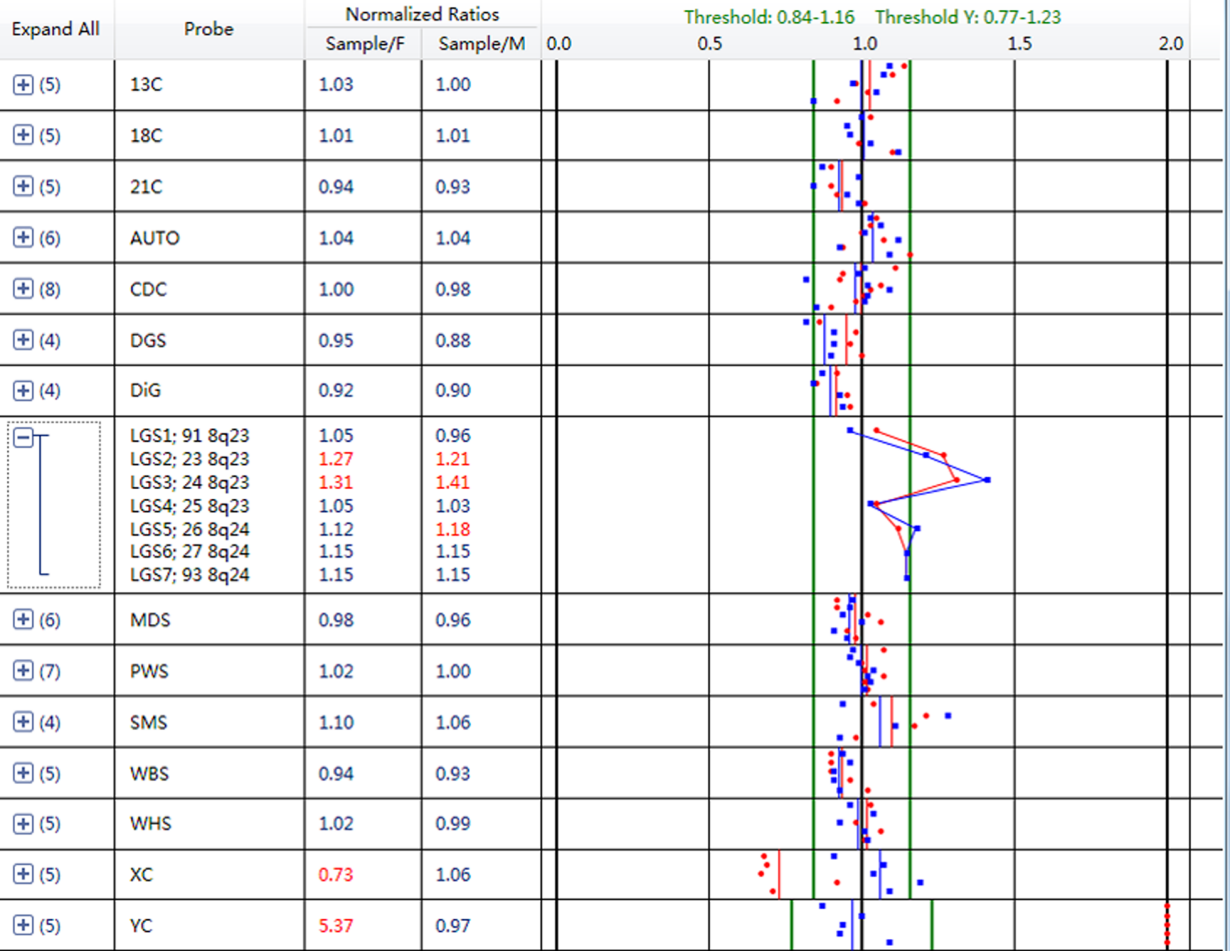
Bobs™ assay of the fetus. Blue dots represent the proportion of tested DNA compared with the male reference DNA. Red dots represent the proportion of tested DNA compared to the female reference DNA. Green lines are the normal range for the signals. Bobs™ assay showed that the signal on chromosome 8 was generally elevated

Karyotyping was performed on cultivated amniocytes. A total of 73 metaphases were analyzed and trisomy 8 was found in 1 metaphase out of them (Fig. 3A), indicating the existence of T8M. As is Fig. 3B, SNP-array on cultivated amniocytes and neonatal cord blood cells showed with ~ 2.1, a slightly higher than the normal signal range (~ 2.0), this suggested that the T8M percentage was indeed around 10%. Then, interphase FISH on the uncultivated neonatal cord blood was subsequently performed to estimate the mosaic level of trisomy 8 (Fig. 3C, D). Fifty interphase cells were counted and five cells were found to carry three red signals, confirming the T8M percentage as 10%. The Bobs™ fluorescence data done on this material also showed a significant signal increase in 8q23-8q24 region (Fig. 4).

### Follow-up

Between 17th and 23rd week of gestation several multidisciplinary consultations were done and pregnant women and her family members were informed that this fetus may have some defects later on; still the family decided to continue the pregnancy until birth at 36th week of gestation. The birth weight was 3,110 g and the baby had APGAR scores of 10 at 1 and 5-min. The child was followed-up when he was 3 years old. He presented periodic fever and language retardation, and manifested an asymmetrical cheek and low-set ears.

## Discussion

T8M is rare, and its prenatal diagnosis is difficult because the trisomy 8 cells may disappear during the culture of amniocytes [9]. T8M has been reported to be missed at amniocentesis by cytogenetic analysis using cultivated amniocytes [10–12]. Hulley et al*.* [13] found that the disappearing of trisomy 8 cell line may be caused by growth disadvantage.

In the current investigation, a risk of fetal trisomy 8 was initially identified by cffDNA. The fetus manifested hydronephrosis and irregular spine on ultrasound and MRI. According to previous reports, patients with T8M may present with bilateral hydronephrosis [14–16]. The fetal karyotype was 47,XY, + 8[1]/46,XY[72]. This imply that if cffDNA screening had not suggested the possibility of fetal T8, the karyotype analysis would likely miss the diagnosis of T8M.

cffDNA, derived from trophoblasts and circulating in the maternal plasma, is used to detect potential fetal chromosomal abnormalities [17]. The analysis of cffDNA with the next-generation sequencing, provides a well-validated method to identify fetal aneuploidies. Liang et al*.* [18] recruited 94,085 pregnant women for a prospective study and showed that cffDNA-based screening for trisomy 21 was superior to any other clinically available screening methods, with a positive predictive values (PPVs) of 95%. For other aneuploidies, such as trisomy 18, trisomy 13, rare trisomies, and sex chromosome aneuploidies, the PPVs are relatively lower compared with trisomy 21, approximately 82%, 46%, 29%, and 47%, respectively.

Identification of RATs by NIPT [5, 19, 20] have low positive predictive values and thus their inclusion in routine testing is under debate [21]. In fact, with an aggregate incidence of approximately 0.3% [19, 22], RATs are common and associations between RATs and feto-placental diseases have been increasingly reported. Even though RATs are in majority of cases restricted to placenta, cases like the present one can also be among those being detected by NIPT. Their detection could help omitting increased risk of miscarriage, intrauterine growth restriction [23, 24], low birth weight [25], small-for-gestational-age infants [26], uniparental disomy [22] and neonatal intensive care unit admission [27]. Therefore, early identification of RATs is helpful to control risks of adverse pregnancy outcomes [28].

However, NIPT results identifying fetal RATs need to be checked by further cytogenomic approaches, like SNP-array, FISH and Bobs™ on fetal cells. Fetal imaging is also necessary to evaluate the presence or absence of structural abnormalities. Finally, follow-up is needed to evaluate the long-term outcomes of babies with RAT mosaicism.

## Data Availability

The datasets used and/or analyzed during the current study are available from the corresponding author on reasonable request.
